# Improving and re-developing the Clerical Resolution Online Widget (CROW): open-source software for clerical review

**DOI:** 10.23889/ijpds.v9i5.2578

**Published:** 2024-09-18

**Authors:** Hannah O'Dair, Sarah Cummins, Ira Ashia, Rachel Huck

**Affiliations:** 1 Office for National Statistics

## Objectives

Clerical review is an integral part of linkage and is fundamental to quality assurance. CROW is a low dependency, adaptable, clerical matching tool that has been deployed across 40 linkage projects. Due to limitations of the interface, with the initial version (including the limited ability to view large clusters); this project sought to re-develop the tool as a flask application.

## Approach

Our approach was to transform the existing CROW desktop application into a Flask (web based) application. Flask was chosen because it is a flexible web framework that enabled the development of a more dynamic interface, as well as better integration with the infrastructure of secure environments. We also engaged with accessibility experts to ensure the inclusivity of our product. Once the development was completed, testing was carried out by a range of users and stakeholders, to identify further issues and bugs for resolution.

## Results

The successful redevelopment of CROW into a Flask application improved functionality, usability, parametrisation, and accessibility of the tool. Feedback from users indicates that because of using CROW, the clerical matching experience has improved vastly. Users reported that CROW increased accessibility, reducing eye strain and fatigue. CROW also increases efficiency and leads to better decision making.

## Conclusion

The successful redevelopment of CROW is a significant improvement to the clerical review process for data linkage projects.

## Implications

CROW is open-source and low dependency so could be adapted to work in most environments. Therefore, it could be adapted and used by the wider data linkage community.

